# Cannabinoids Hyperemesis Syndrome – An Urgent Call for Timely Diagnosis, Management, and Future Directions– A Case Report and Review of the Updated Literature

**DOI:** 10.1192/j.eurpsy.2024.861

**Published:** 2024-08-27

**Authors:** S. Poudel, J. Choudhari, N. Panta, H. Kumar, S. Ahmed, D. Leszkowitz

**Affiliations:** ^1^Department of Research & Academic Affairs, Larkin Community Hospital, South Miami, United States; ^2^Kathmandu Medical College, Kathmandu, Nepal; ^3^Dow University of Health Sciences, Karachi, Pakistan; ^4^Department of Addiction Medicine, Larkin Community Hospital, South Miami, United States

## Abstract

**Introduction:**

Cannabinoid Hyperemesis Syndrome (CHS) is distinguished by a pathognomonic cyclic pattern of hyperemesis characterized by recurring episodes of severe vomiting every few weeks to months, as well as obsessive thoughts and compulsive behavior, such as a proclivity to take frequent hot baths or showers. It is largely accepted as the most commonly used illicit drug in the United States, with estimates ranging from 42% to 46% lifetime consumption. Despite greater awareness of CHS, practitioners continue to lack comprehension, resulting in an unfortunate delay in patient identification and treatment.

**Objectives:**

The aim of this article is to bring attention to CHS in order to enable clinicians, and more specifically, addiction medicine specialists and psychiatrists, to diagnose it as quickly as possible and thus avoid unnecessary additional invasive examinations and investigations. This will save the patient’s time, prevent financial burdens and mental health stresses, and increase their overall quality of life.

**Methods:**

A thorough screening and data extraction of the relevant articles was conducted using PubMed, Cochrane, and Embase. Databases were used to search for articles on CHS published between January 2021 and September 2023, yielding relevant articles. Keywords used were “hyperemesis”, “cyclical vomiting,” “cannabis” and “cannabinoid”.

**Results:**

We present a case of 20-year-old teens who came to emergency with severe dehydration and vomiting of more than 40 episodes at home. He had multiple admissions for abdominal pain, nausea, and vomiting in the past and was evaluated and diagnosed with gastritis, PUD, and H. pylori infection. A more detailed medical history revealed a frequent use of cannabis over the past few years and symptoms manifestation and worsening is associated with the use of cannabis. After the complete cessation of cannabis, there have been no new symptomatic episodes reported in the patient and the patient is stable clinically.

**Conclusions:**

Cannabinoid Hyperemesis Syndrome (CHS) is a serious health hazard that requires immediate discovery and treatment. Despite the widespread use of cannabis, CHS is often misdiagnosed, resulting in unnecessary medical treatments and complications for patients. Given their special knowledge of linking chronic cannabis use to this syndrome, this case report and literature review highlight the critical role of addiction medicine experts and psychiatrists in quickly detecting and treating CHS. Early detection and treatment, particularly complete cannabis abstinence, are critical in alleviating symptoms, minimizing recurrent hospitalizations, and ultimately improving patients’ overall quality of life.

**Disclosure of Interest:**

None Declared

